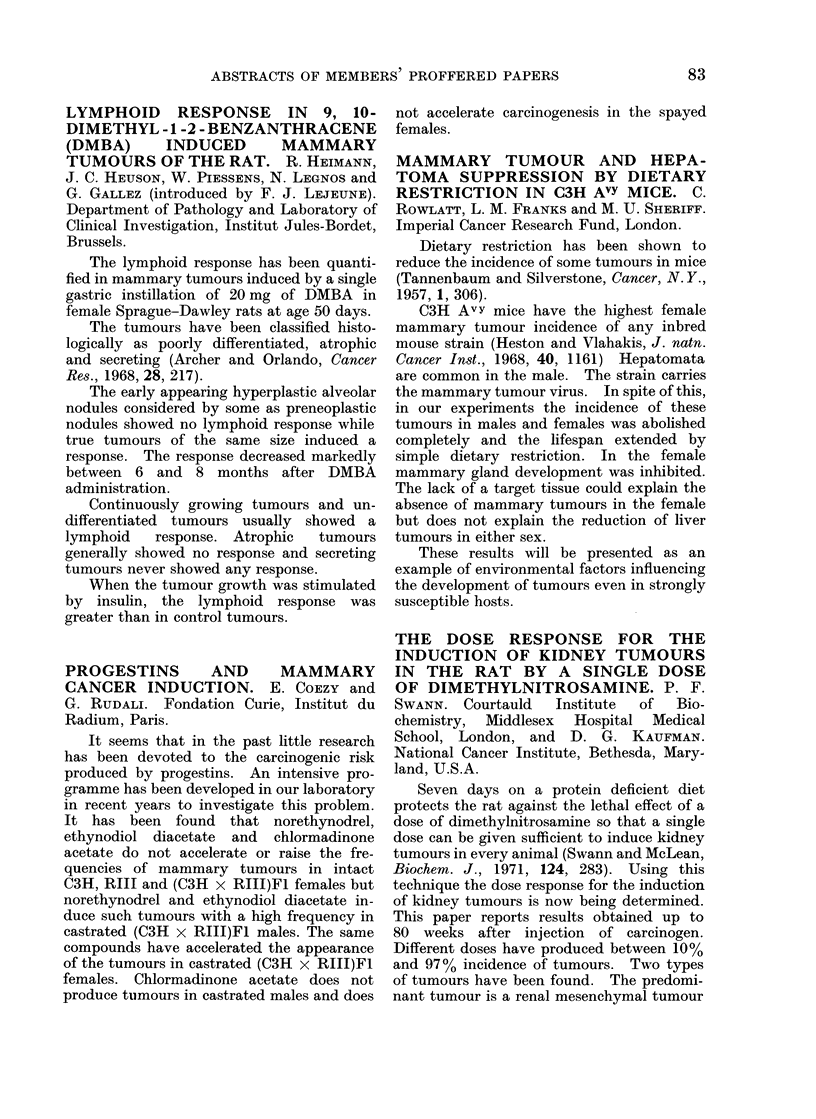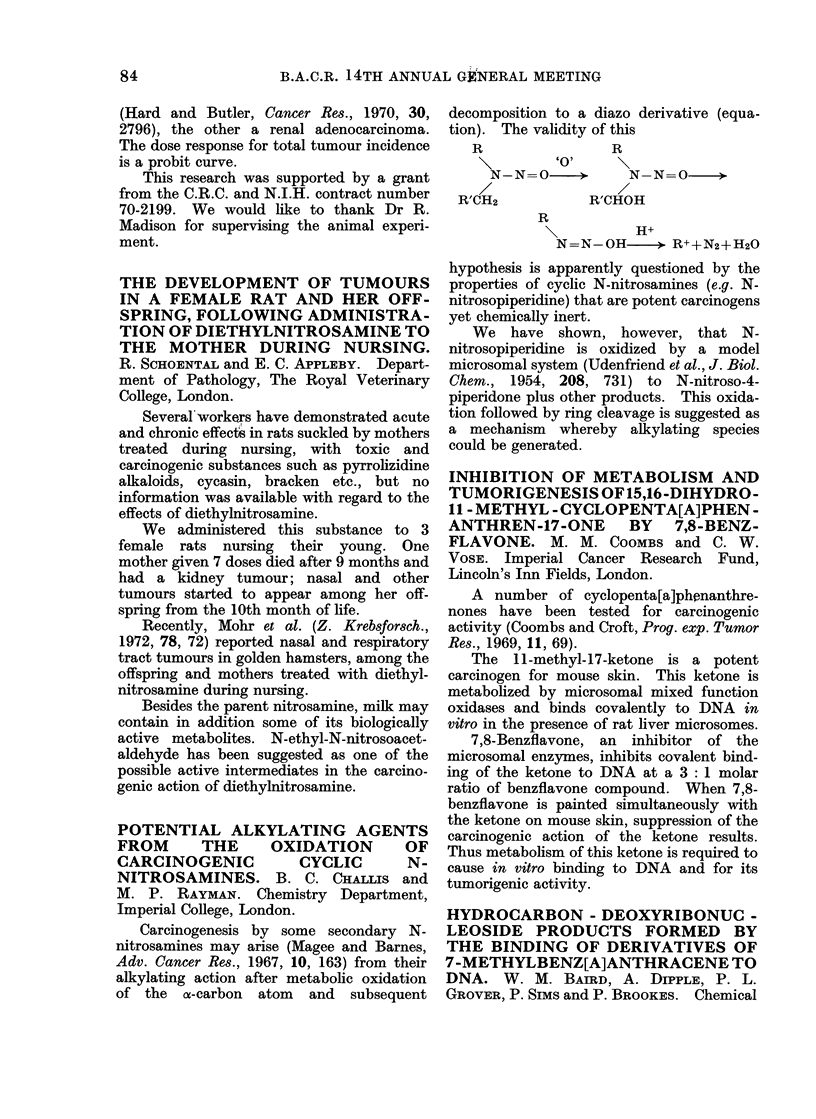# The dose response for the induction of kidney tumours in the rat by a single dose of dimethylnitrosamine.

**DOI:** 10.1038/bjc.1973.99

**Published:** 1973-07

**Authors:** P. F. Swann, D. G. Kaufman


**THE DOSE RESPONSE FOR THE INDUCTION OF KIDNEY TUMOURS IN THE RAT BY A SINGLE DOSE OF DIMETHYLNITROSAMINE.** P. F. SWANN. Courtauld Institute of Biochemistry, Middlesex Hospital Medical School, London, and D. G. KAUFMAN. National Cancer Institute, Bethesda, Maryland, U.S.A.

Seven days on a protein deficient diet protects the rat against the lethal effect of a dose of dimethylnitrosamine so that a single dose can be given sufficient to induce kidney tumours in every animal (Swann and McLean, *Biochem. J.*, 1971, **124**, 283). Using this technique the dose response for the induction of kidney tumours is now being determined. This paper reports results obtained up to 80 weeks after injection of carcinogen. Different doses have produced between 10% and 97% incidence of tumours. Two types of tumours have been found. The predominant tumour is a renal mesenchymal tumour

(Hard and Butler, *Cancer Res.*, 1970, **30**, 2796), the other a renal adenocarcinoma. The dose response for total tumour incidence is a probit curve.

This research was supported by a grant from the C.R.C. and N.I.H. contract number 70-2199. We would like to thank Dr R. Madison for supervising the animal experiment.